# Indents

**Published:** 1909-07-15

**Authors:** 


### INDENTS.

☞**Brief Articles of Technical or Scientific Value will receive notice and comment. Contributions invited.**

*Death of Dr. C. Newlin Pierce.* The many friends, personal and professional, of Dr. C. Newlin Pierce will learn with very great regret that death has closed his long, honorable and useful career.

Entering upon the study and practice of dentistry at an early period in its history as an organized profession, he has been closely identified with, and earnest in his advocacy and support of, every movement promotive of its advancement.

His ability as a practitioner and interest in the science as well as the art of dentistry equipped him in an eminent degree for the educational work to which he was called at an early period in his professional life, and in which he continued almost continuously up to his final retirement from all professional pursuits.

As a teacher he was earnest, painstaking and illuminative, always commanding the respectful attention of his students, while his kindly and sympathetic nature and fatherly interest in their welfare won for him a measure of personal love and esteem not always accorded to a teacher.

Through his rare personal qualities and professional attainments he early became and always remained one of Philadelphia's most successful practitioners. His patients had confidence in his skill, judgment and integrity, as also had his fellow practitioners, by many of whom his advice and cooperation as a consultant in difficult cases were in frequent demand.

Although not a graduate in medicine and fully satisfied to be regarded simply as a well-informed and well-qualified dentist, he had a wide acquaintance with medical literature and kept well in touch with the progress of medical and related sciences.

Notwithstanding his laborious duties as a teacher and practitioner, to which he gave even more than the full measure of his strength, he did not lose interest in the great moral issues of the time, nor neglect the duties of good citizenship, but gave to the cause of good government and of civic and national righteousness faithful cooperation and moral and financial support.

Although his life, so full, rounded and beneficent, has ended, its example will long remain as a living force, an inspiration to others to live like him for worthy aims, faithful to duty and loyal to the profession he served so well and so greatly adorned.—*Dental Brief.*

*Nitrous Oxide Administration and Heart Disease.* In cases of heart trouble I depend upon the general vitality of the patient. The gas does not affect the heart function, and to my mind there is no more fear of the heart stopping under nitrous oxid than there is in natural sleep, except that it may be affected by previous suffering and extreme nervous excitement immediately preceding and during the administration. A heart that has been over-stimulated by nervousness and fear to such an extent as many patients exhibit in anticipation of the taking of an anesthetic must always be approached with extreme caution, and these are cases that must be declined. I had one case of a patient who was a victim to the weakening effects of the grippe upon the heart, to whom I refused to give the gas one afternoon. The next day I received word that he had died. There is no necessity for running such risks as that. It is far more dangerous to give nitrous oxid to an athlete who has taxed his powers to the point of exhaustion and collapse than it is to administer it to a patient whose heart is what we term a bad one, and yet whose nerve and muscle strength have never been abused.

I never use a face-piece. Nitrous oxid, though composed of nitrogen and oxygen, does not give up its oxygen at the temperature of the human body, consequently we have a dual effect produced, one being true anesthesia and the other asphyxia, and there are some cases which will asphyxiate before they will anesthetize.

Narcosis produced by asphyxiation is a very disagreeable exhibition, as well as a dangerous one, therefore I use the mouthpiece. I can keep in view the lips during the whole process of administration, and should asphyxia appear, by raising the lips and allowing a portion of air to pass in with each inspiration the color will be preserved, and perfect anesthesia will be produced. Oxygen combined with nitrous oxide will prove quite as satisfactory, but for the short operation of tooth-extraction I prefer the less complicated operation and the simplicity of the process.

Imperfect breathing is the cause of greater anxiety in administering nitrous oxid than is impaired heart function.

Cases of asthma, goiter, enlarged tonsils, and thickened bronchiae must be watched very carefully, for the reason that when the asphyxiation is rapid and prominent, oxygen is taken up very slowly, and should the respiratory effort become slower or entirely suspended, which effect is produced in narcosis by the want of oxidation, artificial respiration may become necessary.

All these symptoms of danger may be overcome by keeping the color of the blood in view as shown through the membrane of the lips.—Dr. J. D. Thomas in *Cosmos*.

*The Porcelain Inlay.* The inlay process was supposed to be similar to paperhanging. Select a porcelain shade, bake it, cement it in, smile and collect.

I laboriously cut cavities in extracted teeth and went to work. I used all the various porcelain bodies. Some made to fuse on the lighted end of the after-dinner cigar, all the way up to the highest fusing. I also selected porcelain teeth of the proper shade, broke them up in a mortar, ground, strained and filtered; precipitated with H2O and fused. I fused with gas, electricity and profanity. All inlays were to be invisible after three months' wear and the proper solution of the cement film. Only edentulous patients were barred from the joys of esthetic dentistry.

I baked white inlays from bottles marked dark brown, and smoky-colored inlays from white body, and performed many wonderful chromatic stunts.

Later it was announced from Chicago that there had been a mistake made in the calculation of the latitude, and that all cavities should be cut like a scroll-saw puzzle of Rover in the Garden. Then it was proclaimed from the Metropolis of Indiana that before one should select the shades of an inlay he must take the three primary colors and paint the aurora borealis to scale. I bear some scars as the result of this revolution and some my patients bear. As tooth savers my porcelain inlays have been quite successful; but as their chief merit is an esthetic one, I have not attained it. This I ascribe—note the modesty—to two reasons: First, because there is in many mouths a marginal discoloration or deposition following the solution of the cement line. Second, lack of practice. I have worked hard enough on porcelain inlays to be a master of the art, but I am not. The limited number of cavities suitable for them and the limited number of patients in my practice able and willing to pay a commensurate fee for their insertion make it seemingly impossible for me to acquire and keep the necessary technique. One result of the porcelain era has been the more general use of the baked porcelain crowns for incisor and bicuspid teeth. To me it has been a most pleasant and successful solution.

I know it is horribly unfashionable to bake a crown when it may be cast, but I must continue to be unfashionable until another benefactor invents a method of casting porcelain.

The net result to me of this revolution is a strong faith in porcelain inlays in cervical and proximal cavities in incisors not involving the angle or the pulp; a full faith in the baked porcelain crown; an accumulation of varied and sundry porcelain bodies, most of them for sale cheap, and an ability to bear trials, troubles and tribulations with some degree of dignity.—S. H. Voyles in *Dental Brief.*

*Carbolic Acid Gangrene.* Two observers writing from cities so remote from each other as St. Louis and Philadelphia have reported simultaneously five cases of gangrene following the use of carbolic acid as a surgical dressing. J. A. Kelly, in the February number of *Annals of Surgery,* reports a woman, aged 27, who applied a carbolic solution of unknown strength to a punctured wound of the finger. The dressing remained in place only five hours when intense pain brought about its removal. The finger was found to be dead and blackened from gangrene. The patient neglected advice of immediate amputation, but later consented to have the finger removed at the meltatarsophalangeal joint. In *St. Louis Medical Review,* also for February, W. E. Leighton cites four cases of carbolic acid gangrene of fingers and toes. He considers that two factors are productive of this untoward result of the use of carbolic acid: the skin becomes hardened and contracted by the application, and hence produces mechanical constriction after the manner of a very tight bandage; or an artery, already, sclerotic, in the affected digit offers poor resistance against local necrosis.

The faith possessed by the laity in carbolic acid as a disinfectant and germicide leads them very frequently into error. In the first place there are many drugs equally or more powerful as antiseptics which do not have the local anesthetic effect or disagreeable odor of phenol. This anesthetic effect of phenol is one of its greatest dangers, for it is likely that when pain following its use begin in adjacent tissue, irreparable damage has already been done. However, the layman with an injured member, upon visiting a nearby drug store, either demands a carbolic solution or, as quite often happens, is supplied with the drug by the pharmacist, who speaks only of its poisonous constitutional effect and neglects to warn the patient concerning its local action.

There is then, sufficient reason for limiting the sale of this drug to physicians exclusively, and for not allowing it to be dispensed to almost anyone who is willing to sign the "poison book." Most surgeons of experience have seen cases of carbolic gangrene caused either by the patients ignorance, or, in a few cases, by the failure on the part of the physician who first saw the case to remember what may happen. Moreover, it is not the strong, but the weak percentages of the drug that may do damage. A finger has been lost through the use of a ten (10) per cent. carbolic ointment applied to an open wound, and a like result has followed the application of solutions 1 in 60 and 1 in 100 strength, respectively. In most hospitals, especially in dispensary service, carbolic acid is used chiefly for the sterilization of instruments which are washed in 95 per cent. alcohol and sterile water before use. Certainly in the light of past experience it is far safer to apply lead and opium, or alum acetate, or bichlorid of mercury of suitable strength than to take chances with a drug so powerful in its local action as is phenol.—*Medical Record.*

*Medical Education for Dentists not Desirable.* Those of our readers who keep in touch with current dental events will recall that at its last meeting the Virginia State Dental Association placed itself on record as favoring the institution in that State of measures to secure the full recognition of dentists as medical specialists.

For the furtherance of that purpose a special committee was appointed whose initial movement has been the publication of a pamphlet in which, after a brief resume of the history of the organization of the early dental colleges, accompanied by criticism of their management, and followed by a dissertation upon the scope and importance of dental service, the committee recommended that on and after a given date, 1915, all applicants for a license to practice dentistry in Virginia shall be required to pass the full examination in medicine before the regular medical examining board, in addition to an examination on dental subjects, the latter to be in charge of two dental examiners to be added to the medical board.

In the advocacy of this proposed change the dominant thought in the minds of the committee appears to be that dentistry is looked upon by medical men as a calling essentially different from their own—in fact, a trade rather than a profession, for the reason that dentists do not, or need not hold the medical degree, and that just as soon as the medical degree is made obligatory the point of view of the medical world will be entirely changed, and although the dentist's work must remain the same, if it is to be done at all, he will no longer be regarded simply as an artisan, but will be recognized and welcomed as a brother practitioner.

Whether this is or is not a thought dominant in the minds of the committee, it is certainly one much emphasized by them and by those who think with them, and must be regarded as one of the leading reasons for their proposal to make a full medical education obligatory upon all seeking to qualify for dental practice.

Unfortunately, for its value as an argument in favor of the proposal, the assumption on which it is based is not sustained by the facts of dental history. The dentist who, in 1839, as stated by the committee, and others who at later periods vainly thought to have a dental course established as a part of the curricula of medical schools, were almost without exception themselves medical graduates. This was true also of a great majority of the founders of the early dental colleges, and, indeed, of a large percentage of the leading dental practitioners throughout the world; and yet the fact that, according to the standards of their time, they were qualified medical practitioners specializing as dentists failed to secure for them or their profession the results which the committee claim will now follow the possession of similar qualifications by all dental practitioners.

Not only does that contention fail to be sustained by the history of dentistry in Amerca, but also in other countries. In Austria, for example, every operative dentist is required to have the full medical degree; but there is no evidence that the professional and social status of dentistry is higher than in America, or that in professional ability the dentists of Austria are superior to their American confreres, or have contributed more to the advancement of dentistry either as an art or science.

The fact that dentistry is so largely a mechanical pursuit is, however, not the only reason why, as stated by the committee, it is "looked down upon as a separate and inferior calling." General surgery also is mechanical, and at no very remote period was regarded by physicians and by the laity as a calling distinct from and much inferior to that of the healing of diseases by drugs or by other and more occult agencies. General surgery has come to its own, and to-day is the preferred rather than the despised branch of the healing art not only in spite of, but because of its basis in mechanics; the trained mechanical skill, guided by the anatomical knowledge of the operator, enabling him to effect remedial results and save life in many cases which the medicative art of the physician had failed to cure.

General surgery deals with immediately vital tissues and assumes vital responsibilities. Controlling the mechanism of a surgical operation there must be the skilled and unwavering touch; the trained and resourceful intelligence, ready for all emergencies, and a will-power panic-proof in the presence of impending catastrophe.

Some or all of these qualities are in a greater or less degree demanded of the physician and specialist, as well as of the general surgeon, and each branch of the healing art, including dentistry, is esteemed by society just in proportion as such qualities are necessary to its efficiency, and just in proportion, too, as its responsibilities are vital in character or involve the safety of organs or members whose loss would be an overwhelming and irremediable disaster.

Just in proportion as the public are taught to appreciate the value of and importance of the teeth will the man who saves them be held in esteem; but all sense of proportion must be lacking in those who can imagine that the time will ever come when the loss of a tooth or of all the teeth will be regarded as a calamity at all comparable to the loss of sight or hearing, or when the remedial office of the ophthamologist or aurist will not be regarded as more important than that of the dentist.

It may, of course, be urged that the elevation of the professional and social status of the dentist is not the sole reason why, before being admitted to practice, he should be required to pass the state board examination in medicine and general surgery as well as in dentistry; the further reason being that the present system of dental education in independent dental colleges or in the dental departments of medical schools does not in any adequate degree qualify him for the practice of his profession, the logical corollary being that there are at least twenty-five thousand dentists in the United States chargeable to a greater or less degree with professional incompetency.

It will probably be admitted by the committee that a majority of them know fairly well how to treat and fill teeth, regulate them and, in case of necessity, extract them and provide substitutes; but if in addition to these, which are evidently regarded as minor and unimportant duties, he is as stated by the committee, "expected not only to diagnose but to successfully combat the ravages of every constitutional disease," the sooner he qualifies for general practice the better.

A saner view of the dentist's true relation to a constitutional disorder, such as syphilis, for example, would seem to be that when a specific lesion occurs in the mouth the dentist's duty—and it is an important one—ends with the diagnosis, and that as a dentist, or even as a stomatologist he is not called upon to treat the case, but should refer it to the syphilologist or general practitioner.

If, however it is really expected aud required that dentists shall thus invade the field of fieneral practice, then the contention of the committee is unassailable. The prospective dentist must necessarily be a graduate in medicine, with all the knowledge and experience required for the conduct of a medical practice. If after four years of strenuous study in a medical school, supplemented by a year or two of hospital practice, his original purpose has not been abandoned in favor of the larger field upon which his medical diploma and state board certificate entitle him to enter, he can then devote a few months—eight has been suggested—to the study of dentistry.

Regarded as an expedient for lessening competition in dental practice, this educational scheme has the merit of probable effectiveness; but as a means for bringing about that full professional and social recognition for which its authors hope it is quite certain to be futile; while as a standard of requirement for all who enter upon the dental novitiate, including those whose only desire is to qualify themselves for the legitimate duties of legitimate dentistry, and whose only ambition is to perform these duties well, it must be regarded as unreasonably exacting and therefore ill-advised.—Editorial in *Dental Brief.*

